# Vibration Analysis for Fault Detection of Wind Turbine Drivetrains—A Comprehensive Investigation

**DOI:** 10.3390/s21051686

**Published:** 2021-03-01

**Authors:** Wei Teng, Xian Ding, Shiyao Tang, Jin Xu, Bingshuai Shi, Yibing Liu

**Affiliations:** 1 Key Laboratory of Power Station Energy Transfer Conversion and System, North China Electric Power University, Ministry of Education, Beijing 102206, China; bingshuai134@126.com (B.S.); lyb@ncepu.edu.cn (Y.L.); 2 China Green Development Investment Group Co. Ltd., Beijing 100020, China; fd_dingxian@163.com (X.D.); lvfa_yjy@163.com (J.X.); 3 China Resources Power Technology Research Institute Company, Shenzhen 518002, China; tangshiyao3@crpower.com.cn

**Keywords:** wind turbine drivetrain, vibration analysis, fault detection, signal processing, failure mechanism

## Abstract

Vibration analysis is an effective tool for the condition monitoring and fault diagnosis of wind turbine drivetrains. It enables the defect location of mechanical subassemblies and health indicator construction for remaining useful life prediction, which is beneficial to reducing the operation and maintenance costs of wind farms. This paper analyzes the structure features of different drivetrains of mainstream wind turbines and introduces a vibration data acquisition system. Almost all the research on the vibration-based diagnosis algorithm for wind turbines in the past decade is reviewed, with its effects being discussed. Several challenging tasks and their solutions in the vibration-based fault detection of wind turbine drivetrains are proposed from the perspective of practicality for wind turbines, including the fault detection of planetary subassemblies in multistage wind turbine gearboxes, fault feature extraction under nonstationary conditions, fault information enhancement techniques and health indicator construction. Numerous naturally damaged cases representing the real operational features of industrial wind turbines are given, with a discussion of the failure mechanism of defective parts in wind turbine drivetrains as well.

## 1. Introduction

Wind energy has expanded rapidly in the past decade due to its clear and renewable properties. By the end of 2019, over 650 GW of installed capacity of wind energy had been put into operation worldwide [1]. In China, this capacity is 237 GW [1], accompanying over 100,000 wind turbines erected, among which 80% of the power supply from wind energy is contributed by doubly fed wind turbines equipped with gearboxes, and 20% is provided by direct-drive wind turbines without gearboxes.

Suffering from harsh environments, e.g., stochastic wind, large temperature difference and alternating load, mechanical parts (gears and bearings) of drivetrains in industrial wind turbines are prone to failure [2,3,4]. Although their failure rate is lower than electrical parts [4,5], mechanical faults of wind turbines may cause longer downtime, more expensive transportation and hoisting fees, and even adverse social influence. Therefore, studies on the mechanical fault detection of wind turbines attract increasing attention with the aim of scheduling reasonable operation and maintenance plans, or avoiding catastrophic results. Various monitoring techniques have been developed to support defect detection in wind turbine drivetrains, involving debris analysis [6], acoustic emission [7,8], oil analysis [9,10], electrical signal [11,12,13], angular velocity measurement [14] and vibration analysis [13,15,16,17,18,19].

Using the method of vibration analysis, vibration data collected from transducers are analyzed by signal processing approaches; thus, potential mechanical faults can be detected accurately. This is the most popular condition monitoring and fault diagnosis technique employed in wind turbines, especially for rotating parts, e.g., gearbox and bearings. It performs superiorly to other monitoring techniques in fault location and hardware cost. To date, the vibration-based condition monitoring system has been a standard configuration in wind turbines, which provides a convenient way to acquire vibration data and provide proper diagnosis results. Nevertheless, vibration analysis for the fault diagnosis of mechanical components in wind turbines is an arduous task due to the complex mechanical structures of drivetrains, varying operational conditions and large differences in rotational speeds among various parts in wind turbine gearboxes.

This paper conducts a comprehensive investigation of the vibration-based fault diagnosis of industrial wind turbine drivetrains from the perspective of practicality, in light of most of the published literature and our on-site experiences. Section 2 introduces diversified structures of wind turbine drivetrains, and Section 3 summarizes the computing methods of fault characteristic frequencies of the mechanical parts in wind turbines. In Section 4, the configuration of vibration data acquisition system and the layout of accelerometers with specific performances are recommended. Section 5 illustrates common faults in wind turbine drivetrains to provide the fundamental methods of fault diagnosis. In Section 6, several challenging issues and solutions on the fault diagnosis of wind turbine drivetrains are proposed, involving the fault detection of planetary subassemblies in wind turbine gearboxes, fault feature extraction under nonstationary conditions and fault information enhancement techniques. In Section 7, five research needs and future challenges in the vibration analysis of wind turbine drivetrains are presented. Section 8 concludes the paper. Numerous naturally damaged cases are provided to illustrate the fault diagnosis methods and failure mechanism of wind turbine drivetrains. The investigation in this paper enables maintenance strategies to be scheduled reasonably, which can reduce operational cost at wind farms, and even guide the design, manufacturing and installation of wind turbines.

## 2. Structure of Wind Turbine Drivetrain

The horizontal-axis wind turbine is the mainstream type for the power generation of wind energy, which can be categorized into two types on the basis of whether a speed-up gearbox is equipped or not. One is the doubly fed wind turbine (DFWT), and the other is the direct-drive wind turbine (DDWT).

### 2.1. Drivetrain of Doubly Fed Wind Turbine

**Figure 1:**
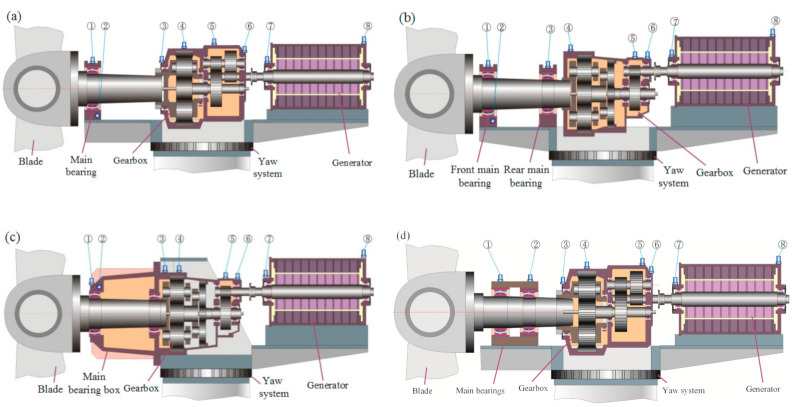
Drivetrains of doubly fed wind turbines: (**a**) independent front bearing pedestal, and rear bearing in gearbox; (**b**) independent pedestal for both front and rear bearing; (**c**) main bearing box and gearbox are combined together; (**d**) front bearing and rear bearing share one pedestal.

In DFWT, the stator windings of the generator are directly connected to a power grid, and the rotor windings of the generator are connected to a power grid through a converter. The slip power between the rotor and stator is adjusted by the converter according to the rotational speed of the rotor. Since the rated rotational speed of the generator rotor is generally over the synchronous speed of the stator current, it is necessary to equip a speed-up gearbox to convert the lower speed of the rotor hub to the higher speed of the generator rotor. In light of the placement of the main bearings and speed-up gearbox, drivetrains of the DFWT can be categorized into the following four types, as shown in Figure 1.

(1) In Figure 1a, the front main bearing is independent, but the rear one is enclosed in the speed-up gearbox. The drivetrain is supported by the front main bearing and two torque arms in the front of the gearbox. This type assures compact structure and sufficient distance between the two main bearings, which may reduce the pressure of the front main bearing. This structure is widely adopted in large-scale wind turbines. The disadvantage of this structure is that the main shaft and gearbox need to be hoisted simultaneously during an installation or replacement.
(2) In Figure 1b, both the front and rear main bearings are independent of the gearbox. To bear the bending moment from blades, the distance between the two main bearings should be long enough, which may enhance the size of the drivetrain. Since the main shaft and gearbox are separated, hoisting can be separately implemented, enabling convenience in maintenance.
(3) In Figure 1c, both the front and rear main bearings are enclosed by a bearing box, which is connected with the gearbox. This structure helps to reduce the length of the cantilever of the rotor hub but enhance the difficulty of maintenance. For example, the precondition of repairing a gearbox is to dismantle the main bearing box. On the other hand, the assembly of the gearbox and generator does not refer to the same benchmark, leading to obvious misalignment between the high-speed shaft of gearbox and the shaft of generator. The bearings on the high-speed shaft of the gearbox and the bearing at the drive end of generator are damageable in this structure.
(4) In Figure 1d, the front and rear main bearings are mounted in one bearing pedestal. The two main bearings need to sustain a large bending moment, thus leading them to be prone to failure. This structure is utilized by early wind turbines whose rated power is less than 1 MW. After over a decade of operation, the main bearings in this structure should be paid more attention.

### 2.2. Drivetrain of Direct-Drive Wind Turbine

In the DDWT, the rotor hub and generator are directly connected, without a speed-up gearbox. The frequency-varying electrical energy from the stator windings of the generator is converted into direct current by a full-power converter, and then inverted into the constant frequency of power grid. The DDWT could be electrically or permanent-magnet excited. Due to the absence of the speed-up gearbox, the number of the mechanical components in the DDWT is substantially reduced in contrast to the DFWT. The main bearings become the merely potential fault sources. Nevertheless, the demagnetization in the permanent-magnet excited DDWT still causes debate among wind turbine manufacturers. Many of them prefer the DFWT rather than DDWT, especially for the selection to be applied in offshore wind farms.

**Figure 2:**
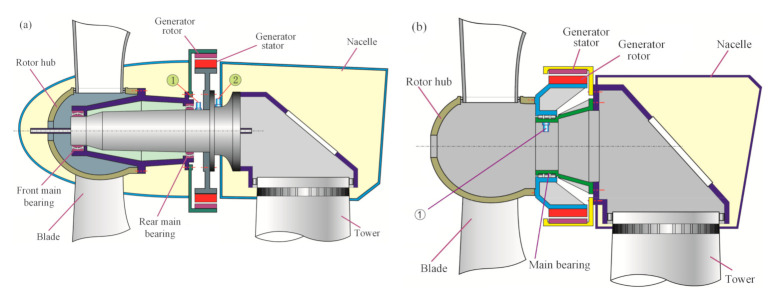
Drivetrains of direct-drive wind turbine: (**a**) external rotor; (**b**) internal rotor.

The mainstream DDWT can be categorized into the following two types, as shown in Figure 2.

(1) In Figure 2a, there are two main bearings supporting the rotor system consisting of the blades, rotor hub, connector and generator rotor. The generator rotor is outside of the generator stator. Generally, the front bearing is a double row tapered roller bearing, and the rear one is a cylindrical roller bearing. The DDWT with an external rotor has higher energy density than the other generators [20].
(2) In Figure 2b, the generator rotor is covered by the generator stator, named the internal rotor DDWT. Only one bearing is adopted to support the heavy rotor system.

The rated rotational speed of the DDWT is much lower than that of the generator of the DFWT due to the absence of a speed-up gearbox. Therefore, the number of pole pairs in the direct-drive generator should be enough to achieve a relatively high frequency of power generation, which enhances the weight and volume of the DDWT. Consequently, the heavy rotor system and stochastic wind impact cause a challenge for the safety operation of the main bearings in the DDWT [21].

## 3. Fault Characteristic Frequency in Wind Turbine Drivetrain

When faults arise on a gear, a phenomenon emerges, which is described as the rotational frequency of the shaft with the faulty gear that modulates the mesh frequency of gear pair or the natural frequency of the gearbox. For parallel-stage transmission, the rotational frequency of the shaft with faulty gear and its harmonics are the fault characteristic frequencies of the faulty gear. There are two types of wind turbine gearboxes equipped in wind turbines: one planetary stage combined with two parallel stages, shown in Figure 1a,d; two planetary stages combined with one parallel stage, shown in Figure 1b,c.

### 3.1. One Planetary Stage Combined with Two Parallel Stages

**Figure 3:**
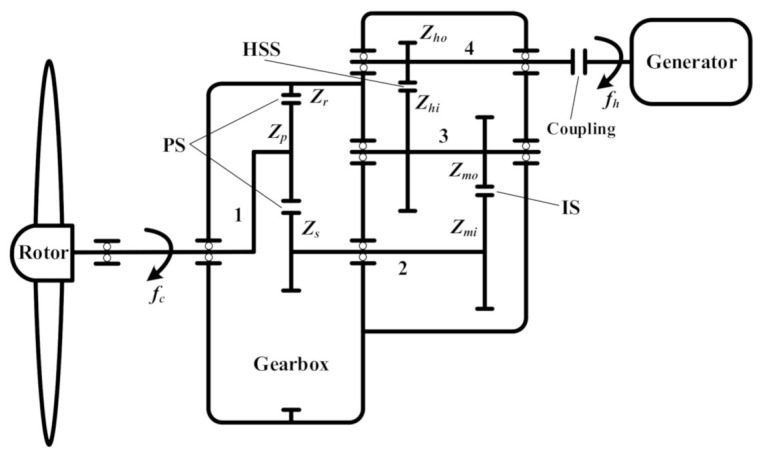
Structure of wind turbine gearbox with one planetary stage and two parallel stages: 1—planet carrier of planetary stage; 2—sun shaft; 3—intermediate shaft; 4—high-speed shaft.

As shown in Figure 3, the gearbox consists of one planetary stage and two parallel stages. The rotor hub is connected to the planet carrier of the planetary stage, and its rotational frequency is denoted by *f_c_*. The three planet gears *Z_p_* mesh with the ring gear *Z_r_* and sun gear *Z_s_*, grouping the planetary stage (PS). The sun gear drives the sun shaft, and further compels the wheel *Z_mi_* to rotate. The sun gear and wheel *Z_mi_* are installed on the sun shaft, sharing the same rotational frequency *f_s_*. The wheel *Z_mi_* and pinion *Z_mo_* mesh with each other and form the intermediate stage (IS). The pinion *Z_mo_* and wheel *Z_hi_* are installed on the same shaft, named the intermediate shaft, with the rotational frequency *f_i_*. The wheel *Z_hi_* and pinion *Z_ho_* group the high-speed stage (HSS). The pinion *Z_ho_* is installed on the high-speed shaft with the rotational frequency *f_h_*. The high-speed shaft is coupled with the shaft of the generator.

The mesh frequencies of different stages and the rotational frequencies of different shafts are computed as in Table 1 and Table 2.

The total transmission ratio of this kind of gearbox is computed as

(1) $$r_{1}=\left(1+\frac{Z_{r}}{Z_{s}}\right)\cdot \frac{Z_{mi}}{Z_{mo}}\cdot \frac{Z_{hi}}{Z_{ho}}$$

During the analysis of vibration signal, the rotational frequency of the high-speed shaft is more easily detected than that of the planet carrier due to the larger vibration energy in the high-speed stage. Thus, the mesh frequencies in Table 1 and rotational frequencies in Table 2 can be reversely deduced from the rotational frequency of the high-speed shaft. The fault characteristic frequencies of *Z_mi_*, *Z_mo_* and *Z_hi_*, *Z_ho_* can be, respectively, denoted by *f_s_*, *f_i_*, and *f_h_*, as in Table 2.

### 3.2. Two Planetary Stages Combined with One Parallel Stage

**Figure 4:**
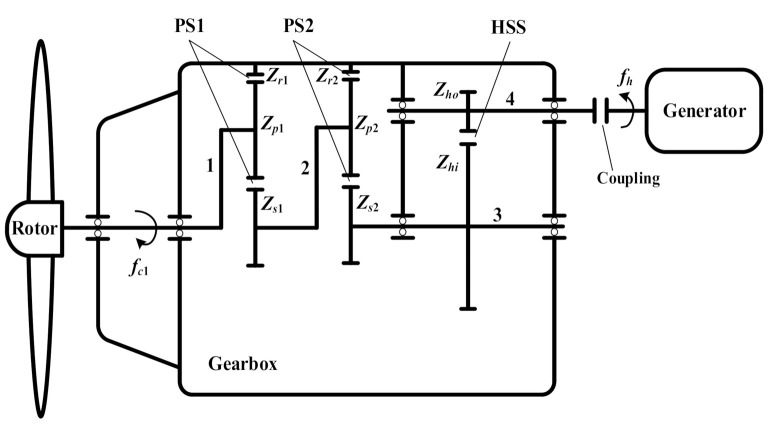
Structure of wind turbine gearbox with two planetary stages and one parallel stage: 1—planet carrier of the first planetary stage; 2—planet carrier of the second planetary stage; 3—the second sun shaft; 4—high-speed shaft.

In order to enhance the transmission ratio at compact nacelle, two planetary stages and one parallel stage are combined to design the wind turbine gearbox. As shown in Figure 4, the rotor hub is connected to the planet carrier of the first planetary stage, and the rotational frequency of the first planet carrier is denoted by *f_c_*_1_. The three planet gears *Z_p_*_1_ mesh with the ring gear *Z_r_*_1_ and sun gear *Z_s_*_1_, grouping the first planetary stage (PS1). The sun shaft of the first planetary stage is connected to the planet carrier of the second planetary stage, sharing the same rotational frequency *f_c_*_2_. The three planet gears *Z_p_*_2_ mesh with the ring gear *Z_r_*_2_ and sun gear *Z_s_*_2_, grouping the second planetary stage (PS2). The rotational frequency of the sun shaft of the second planetary stage is denoted by *f_s_*_2_. The sun gear *Z_s_*_2_ and the wheel *Z_hi_* are installed on the sun shaft of the second planetary stage. The wheel *Z_hi_* and pinion *Z_ho_* form the high-speed stage. The pinion *Z_ho_* is installed on the high-speed shaft with the rotational frequency *f_h_*.

Then, the mesh frequencies of different stages and the rotational frequencies of different shafts in the gearbox with two planetary stages and one parallel stage are computed as in Table 3 and Table 4.

The total transmission ratio of this structure of wind turbine gearbox is

(2) $$r_{2}=\left(1+\frac{Z_{r1}}{Z_{s1}}\right)\cdot \left(1+\frac{Z_{r2}}{Z_{s2}}\right)\cdot \frac{Z_{hi}}{Z_{ho}}$$

The fault characteristic frequencies of *Z_hi_* and *Z_ho_* are, respectively, denoted by *f_s_*_2_ and *f_h_* in Table 4.

### 3.3. Fault Characteristic Frequency in Planetary Stage

**Figure 5:**
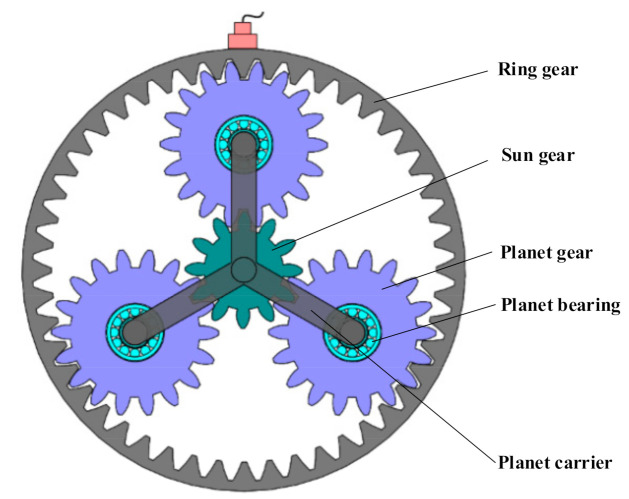
Structure of the planetary stage.

For the gear transmission in the planetary stage, the planet gears have both revolution and autorotation motions. Thus, the computation of the fault characteristic frequency of the planetary stage is different from that of parallel stage. The structure of the planetary stage is shown in Figure 5, which consists of the ring gear, sun gear, planet gears, planet carrier and planet bearings. The ring gear is stationary with an accelerometer mounted on the outer surface to monitor the health status of the planetary subassemblies.

Vicuña proposed a theoretical vibration model for a planetary gearbox only considering the planet-ring meshing processes and the ring gear as the transmission path [22]. Koch and Vicuña pointed out that the phenomenological model coincided better with the Fourier spectrum of vibration signal than the dynamic model for the planetary gearbox [23]. The lumped-parameter model and phenomenological model were compared in [24], which showed that they were both in agreement with experimental measurements with ring gear fault, planet gear fault and sun gear fault. Manufacturing errors of gears could be regarded as one type of distributed fault in planetary gearboxes. Inalpolat and Kahraman [25] developed a dynamic model to predict modulation sidebands of a planetary gear set with manufacturing errors. Chaari et al. [26] analyzed the influence of eccentricity and profile error on the dynamic behavior of planetary gears. They also investigated the influence of tooth pitting and cracking on gear mesh stiffness [27]. Feng and Zuo [28] proposed a vibration model of a planetary gearbox considering three transfer paths and gave the fault characteristic frequencies of planet gear, sun gear and ring gear. Lei et al. [29] established a phenomenological model of a planetary gear set with the consideration of all the time-varying vibration transfer paths from gear meshing points to a fixed transducer. Liang et al. [30] developed a vibration signal model of a planetary gear set by incorporating the effect of multiple vibration sources and a changing transmission path. Chen and Shao [31] simulated the mesh stiffness with the growth of crack size of the ring gear.

In summary, when a fault arises on the planet gear, there is an impulse during its meshing with the sun gear and ring gear, respectively. When there is a fault on the sun gear, a shock arises during its meshing with each planet gear. When a fault arises on the ring gear, there will be an impulse when each planet gear passes the defective location of the ring gear. Thus, the fault characteristic frequency of the planetary stage is deduced in Table 5.

Where $f_{p}^{\left(p\right)}$, $f_{s}^{\left(p\right)}$ and $f_{r}^{\left(p\right)}$ are the fault characteristic frequencies of the planet gear, sun gear and ring gear, respectively. $f_{p}^{\left(p\right)}$ in Table 5 is also the rotational frequency of the planet gear with respect to the planet carrier. For local faults on the planet gear, the fault characteristic frequency $f_{p}^{\left(p\right)}$ might be 2*f_PS_*/*Z_p_*, because of the twice shocks from planet gear meshing with the ring gear and sun gear during one rotation of the planet gear relative to the planet carrier. The 3 in Table 5 means there are three planet gears in the planetary stage of the wind turbine gearbox. It is noteworthy that the fault characteristic frequencies of the planet gear and sun gear might be the combination type in the right column in Table 5, because the rotation of the planet carrier and sun shaft generates a modulation effect to the accelerometer fixed on the outer surface of the ring gear when faults emerge on the planet gear and sun gear. *k* and *n* are both integers.

### 3.4. Bearing Supporting Fixed-Axis Gear or Rotor

Cylindrical roller bearing, angular contact ball bearing and deep groove ball bearing are widely applied in wind turbine gearboxes and drivetrains. For any bearing supporting fixed-axis gear or rotor, it is common to compute the fault characteristic frequency as in Table 6.

Where *f_r_* is the rotational frequency of the shaft assembled with the rotating part of the bearing (mainly inner race), *d* is the diameter of rolling element, *D* is the pitch diameter of the bearing, *N_b_* is the number of rolling elements and *φ* is the bearing contact angle. If the outer race of the bearing is revolving, such as the structure of DDWT in Figure 2 and the planet bearings in Figure 5, *f_r_* is the rotational frequency of the rotor assembled with the outer race, and the fault characteristic frequency of the bearing cage is computed as Equation (3).

(3) $$f_{c}^{\left(b\right)}=\frac{f_{r}}{2}\left(1+\frac{d}{D}\cos\varphi \right)$$

### 3.5. Planet Bearings

The bearings supporting planet gears in the planetary stage, named planet bearings, are significant to guarantee the average-load revolution of the planetary gear transmission. The outer race of the planet bearing is fixed with the planet gear, rotating as the self-rotational frequency of planet gear. The inner race of the planet bearing is fixed with the planet carrier. The fault characteristic frequency of planet bearing is shown in Table 7.

Where $f_{p}^{\left(p\right)}$ is the rotational frequency of planet gear with respect to the planet carrier, and the fault characteristic frequency of the planet gear with distributed fault. Due to the additional modulation effect, the fault characteristic frequency of planet bearing may be the combination type in the right column of Table 7.

## 4. Vibration Data Acquisition and Analysis System

The vibration-based condition monitoring system (CMS) for wind turbine drivetrains can be classified into online CMS and portable CMS.

### 4.1. Online Condition Monitoring System

**Figure 6:**
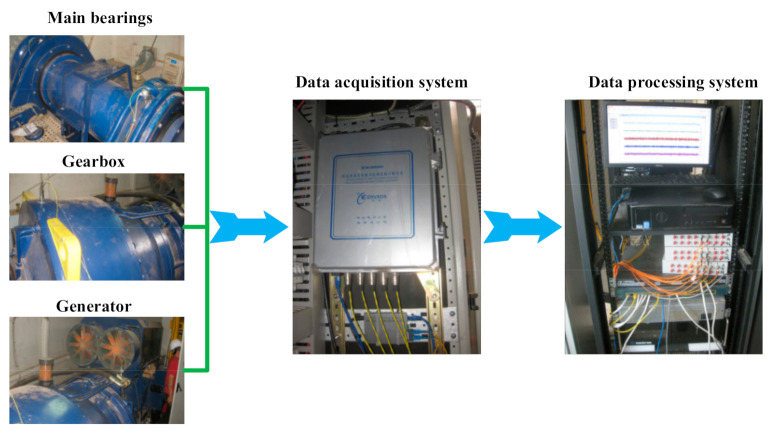
Online vibration data acquisition and analysis system for wind turbine drivetrain.

Online CMS consists of transducers (accelerometers and tachometers), a data acquisition system and a data processing system, which is shown in Figure 6. Several accelerometers are mounted onto the surface of the drivetrain to monitor the health status of the mechanical parts, as shown in Figure 1 and Figure 2. For the DFWT, eight accelerometers are utilized to monitor the main bearings, gearbox and generator bearing. For the DDWT, one or two accelerometers are installed for the condition monitoring of the main bearings. With the tachometer, the rotational frequency of the generator can be accurately measured, although it enhances the cost of CMS. However, it is optional because the rotational frequency of the generator can be estimated through analyzing the vibration signals. The data acquisition system uses multichannel A/D modules to convert analog vibration voltage into digital signal, and implements anti-aliasing filtering for the signal. Many post-processing methods, including time analysis, frequency analysis and time-frequency analysis, are implemented in the data processing system, which can provide the fault diagnosis results of the mechanical parts of the monitored wind turbine drivetrains.

### 4.2. Portable Vibration Data Acquisition System

**Figure 7:**
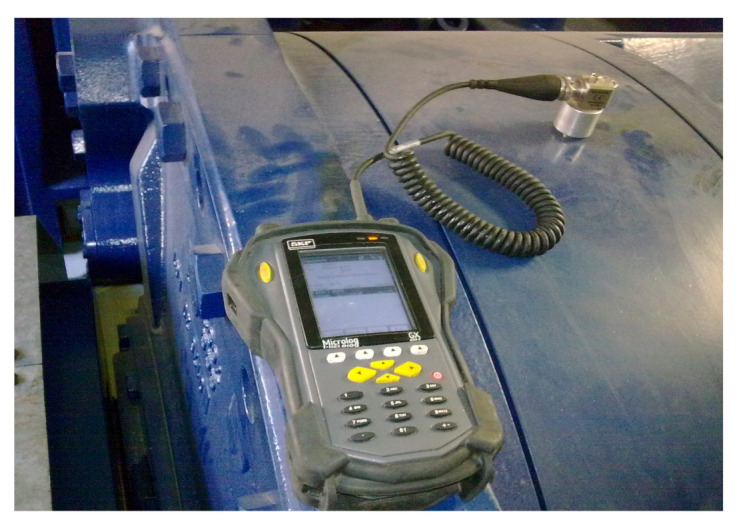
Portable data acquisition system for wind turbine drivetrain.

In order to reduce the cost of the condition monitoring of wind turbine drivetrains, many wind farms utilize portable data acquisition systems for collecting vibration signals point by point, as shown in Figure 7.

The deficiencies of the portable system are obvious: (1) the inefficiency of signal collection and analysis; (2) data acquisition needs human assist; (3) it is hard to compare the vibration features between different channels due to the potential varying rotational speeds at different testing time; (4) power generation loss is inevitable because wind turbines need shutdown when climbing towers. Therefore, online CMS is recommended to be installed for the purpose of the condition monitoring of wind turbine drivetrains.

### 4.3. Layout of Accelerometers

Generally, eight accelerometers are installed on the drivetrain, amongst which two of them are for the main bearings, four of them for the gearbox and two accelerometers for generator bearings. The main bearings and planetary gears operate under low rotational speed and heavy load, so the sampling frequencies for these parts should be low (5120 Hz is suggested), and their sampling durations should be long enough to capture fault characteristics. For example, provided that the rotational frequency of the planet carrier is 0.33 Hz, the sampling duration is suggested to be at least 16 s to capture more than five vibration waveforms to exhibit potential fault information. From the sun shaft to the high-speed shaft, the rotational frequencies increase gradually, leading to an increment in the fault characteristic frequencies of the related gears and bearings. The sampling frequencies for these parts should be high (25,600 Hz is suggested). The sampling durations for these parts can be shortened; generally, 4 s vibration signal is enough to analyze the fault characteristics. The layout of accelerometers of the vibration monitoring system, the sampling frequencies and sampling durations for DFWT drivetrains are suggested in Table 8.

The frequency response range 0.1~5000 Hz and sensitivity of 500 mV/g determine a low-frequency accelerometer, which is suitable for the condition monitoring of the main bearings and planetary subassemblies in front of the wind turbine gearbox. In contrast, the high-frequency accelerometer with a frequency response range 0.5~8000 Hz and sensitivity of 100 mV/g is utilized to monitor the parts from the sun shaft to the generator.

For the DDWT, the supporting part is the main bearing; thus, only low-frequency accelerometers are used for condition monitoring.

## 5. Common Mechanical Faults in Wind Turbine Drivetrains

In this section, several common mechanical faults which can be detected by simple time analysis or envelope analysis are introduced, with their vibration mechanisms being discussed. These fault cases are all from on-site wind turbines, which can provide an empirical guide for the fault diagnosis of the mechanical components in wind turbines.

### 5.1. Faulty Main Bearing of DFWT

**Figure 8:**
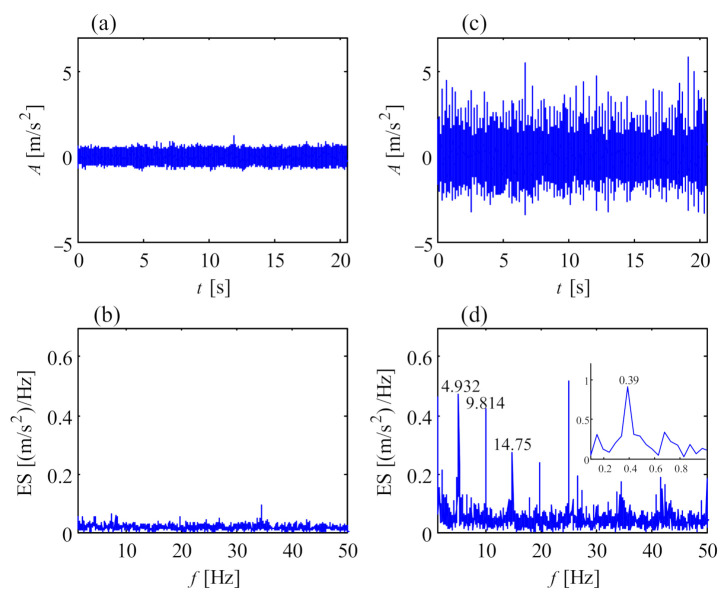
Vibration signal of healthy and faulty main bearing: (**a**) temporal signal of healthy bearing; (**b**) envelope spectrum of healthy bearing; (**c**) temporal signal of faulty bearing; (**d**) envelope spectrum of faulty bearing.

**Figure 9:**
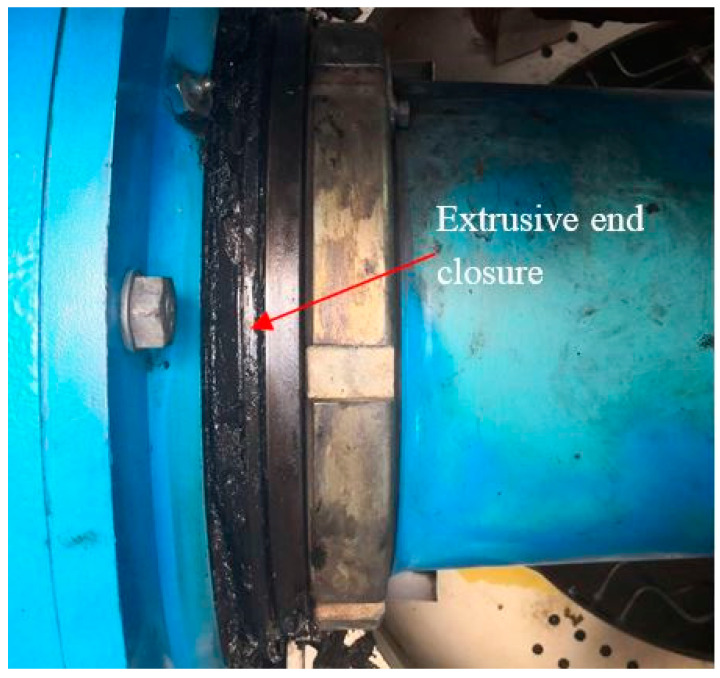
Extrusive end closure of the faulty main bearing.

Figure 8 shows the vibration signals from a main bearing of a 1.5 MW wind turbine. The data were from transducer 1 in the structure of Figure 1b, collected by an online condition monitoring system. Figure 8a is the vibration signal from a healthy main bearing, and Figure 8c is the vibration signal from the same main bearing under fault conditions. Obviously, there are regular shocks in Figure 8c, exhibiting a potential fault of the main bearing. It can be observed that the 4.932 Hz and its harmonics are distinct in the envelope spectrum (ES) [32] of Figure 8d. Dividing the rotational frequency by 0.39 Hz of the main shaft, the fault characteristic of the outer race (4.932/0.39 = 12.65) of the main bearing (SKF240/630ecj) is obtained, deducing that some severe faults emerge on the outer race. The faulty main bearing has already extruded the end closure, as shown in Figure 9.

### 5.2. Faulty Gears in Parallel Stages of Wind Turbine Gearboxes

**Figure 10:**
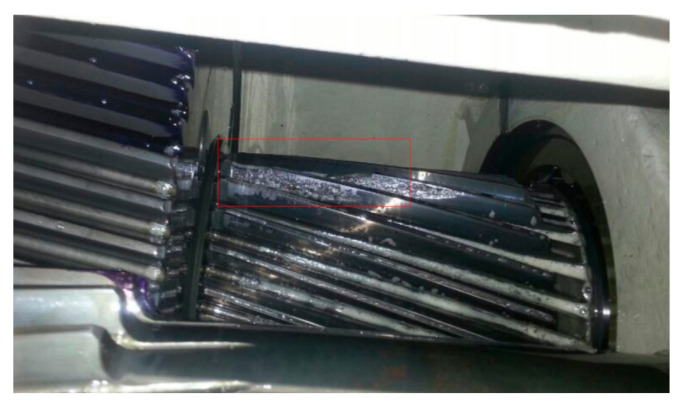
Chipped pinion on intermediate shaft of a wind turbine gearbox.

**Figure 11:**
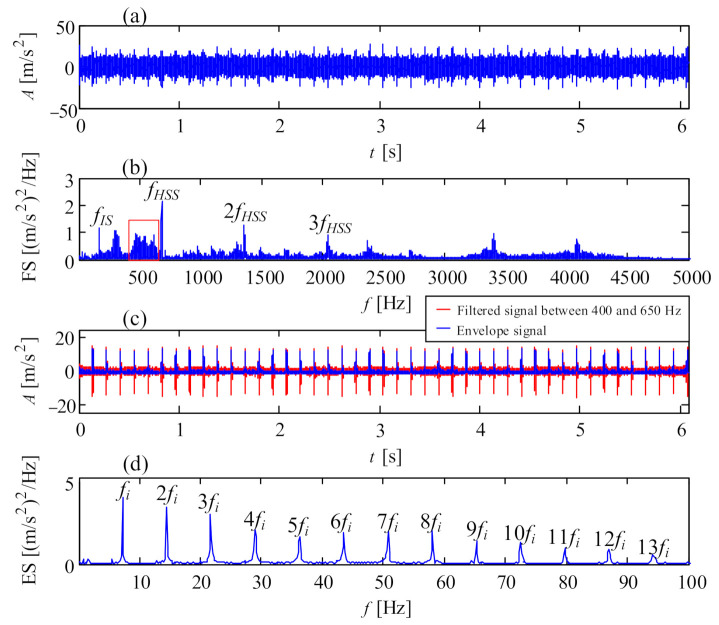
Vibration signal of the chipped pinion on intermediate shaft: (**a**) temporal signal; (**b**) Fourier spectrum; (**c**) filtered signal and envelope signal; (**d**) envelope spectrum.

Since wind turbine gearboxes suffer complex loads and varying rotational speed, some gear faults, such as pitting, chipped tooth or broken tooth, happen frequently. Fortunately, faulty gears in parallel stages are prone to being detected in light of their high vibration energy under high rotational speed. Figure 10 shows a picture of a chipped pinion on the intermediate shaft of a wind turbine gearbox. The vibration signal of this faulty gear is shown in Figure 11a, collected from transducer 5 in the structure of Figure 1a with a portable vibration data acquisition system. The Fourier spectrum (FS) is shown in Figure 11b where the mesh frequency *f_IS_* of the intermediate stage and *f_HSS_* of the high-speed stage occupy the leading positions. Meanwhile, some resonance frequency bands emerge. In Figure 11a, there are acute shocks in the original vibration signal, denoting the local fault on the meshed gear. The signal between 400 and 650 Hz is firstly filtered, then the filtered signal is shown as the red line in Figure 11c, and its envelope signal is shown as the blue line. The envelope spectrum of the filtered signal is shown in Figure 11d where the rotational frequency *f_i_* of the intermediate shaft dominates the whole envelope spectrum. The fault characteristic frequency *f_i_* is in accordance with the faulty pinion on the intermediate shaft.

**Figure 12:**
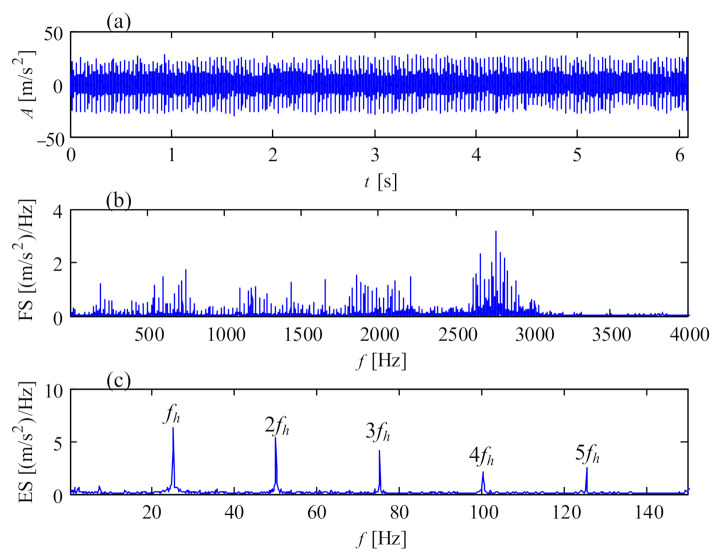
Vibration signal of the broken pinion on high-speed shaft: (**a**) temporal signal; (**b**) Fourier spectrum; (**c**) envelope spectrum.

**Figure 13:**
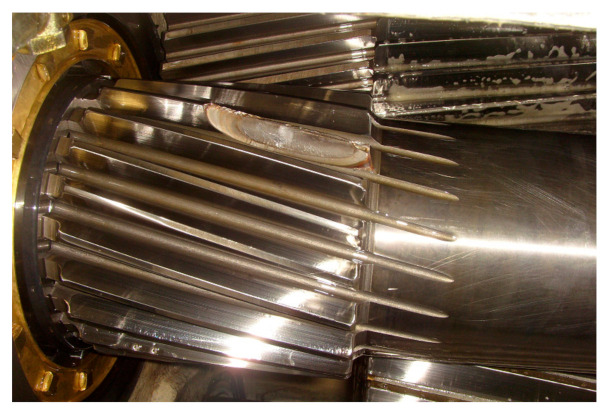
Broken pinion on high-speed shaft of a wind turbine gearbox.

Compared with the vibration signal of the faulty gear on the intermediate shaft, the shocks of the faulty pinion on the high-speed shaft in Figure 12a perform more intensive due to the higher rotational speed of the high-speed shaft. Figure 12b is the Fourier spectrum of the vibration signal in Figure 12a, showing multiple harmonics of the rotational frequency of the high-speed shaft. Figure 12c is the all-pass envelope spectrum of the vibration signal, where clear rotational frequencies of the high-speed shaft are displayed. The broken pinion on the high-speed shaft corresponding with the vibration signal in Figure 12 is shown in Figure 13.

### 5.3. Bearing Fault in the Generator of DFWT

**Figure 14:**
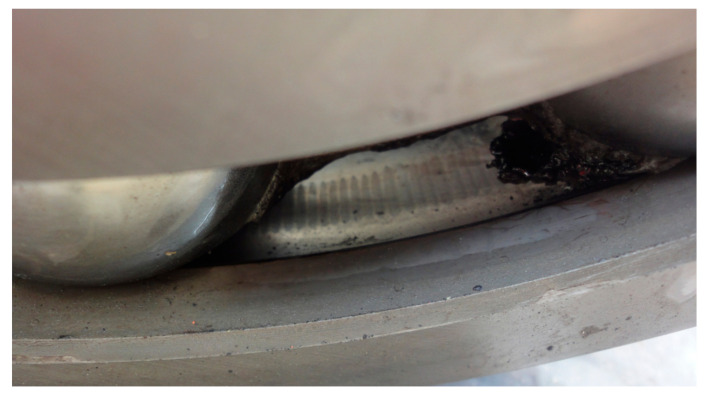
Electric corrosion of a generator bearing.

**Figure 15:**
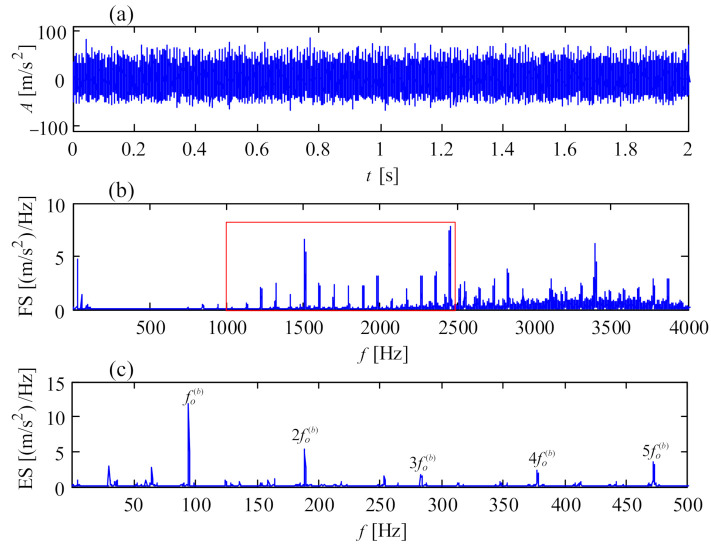
Vibration signal of faulty generator bearing at drive end: (**a**) temporal signal; (**b**) Fourier spectrum; (**c**) envelope spectrum.

Bearing failures in wind turbine generators are common due to their high rotational speed, latent misalignment with the gearbox, induced shaft current, insufficient lubrication, etc. Figure 14 shows an electric corrosion fault of a generator bearing caused by shaft current, and the corresponding vibration signal is shown in Figure 15a. The vibration signal was collected from transducer 7 in the structure of Figure 1c at the drive end of the generator, with a portable vibration data acquisition system. The vibration amplitude exceeds the vibration criterion of VDI 3834 [33], indicating the unhealthy status of the tested bearing. Figure 15b is the Fourier spectrum of Figure 15a, accompanied by a series of spectral peaks from 1000 to 4000 Hz. Demodulating the vibration signal from 1000 to 2500 Hz, the envelope spectrum is obtained, as shown in in Figure 15c, where the fault characteristic frequencies of outer race of the bearing are dominant. These fault characteristic frequencies are in accordance with the faulty bearing in Figure 14.

### 5.4. Bearing Looseness

**Figure 16:**
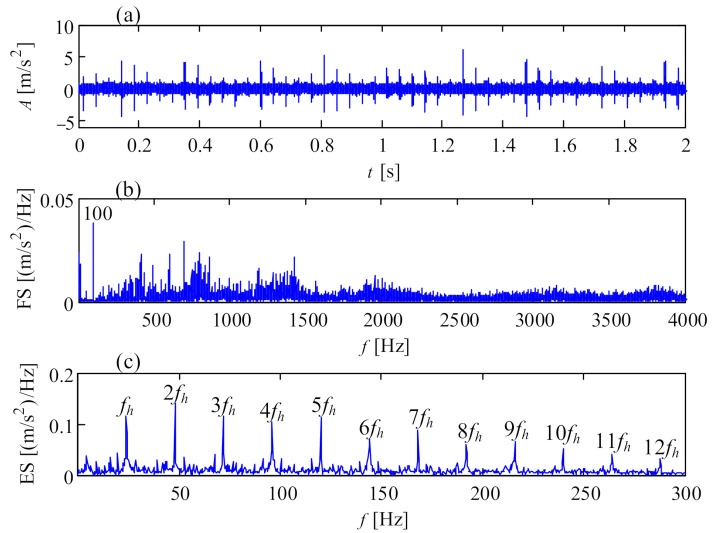
Vibration signal of generator bearing looseness at nondrive end: (**a**) temporal signal; (**b**) Fourier spectrum; (**c**) envelope spectrum.

**Figure 17:**
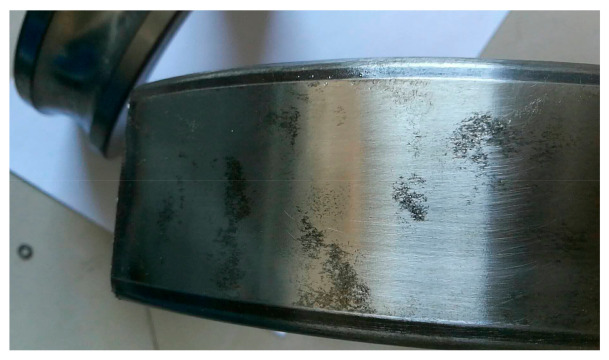
Scuffing on the outer race of a generator bearing due to looseness.

Bearing looseness is a universal phenomenon in wind turbine drivetrains, especially for the bearing on the high-speed shaft of a gearbox or the generator bearings with light radial loads. Looseness is a complex dynamic action which may cause secondary damage to a wind turbine drivetrain, referring to problems such as nonlinear vibration, friction and superheating. Figure 16 is the vibration signal of outer race looseness of a 2 MW generator bearing. The vibration signal was collected from transducer 8 in the structure of Figure 1a, by an online condition monitoring system. In the temporal signal of Figure 16a, each rotation of the generator shaft forms an acute shock, indicating a rub between the outer race and bearing housing during one rotation of the generator shaft. In addition to the electromagnetic interference of 100 Hz, the vibration energy covers almost all of the frequency band, and the envelope spectrum is shown in Figure 16c. In Figure 16c, the rotational frequencies of the generator shaft extend over ten-order harmonics, in accordance with the fault characteristic of bearing looseness [34]. There is obvious scuffing on the outer race of the generator bearing, as shown in Figure 17.

### 5.5. Insufficient Lubrication of Generator Bearings

**Figure 18:**
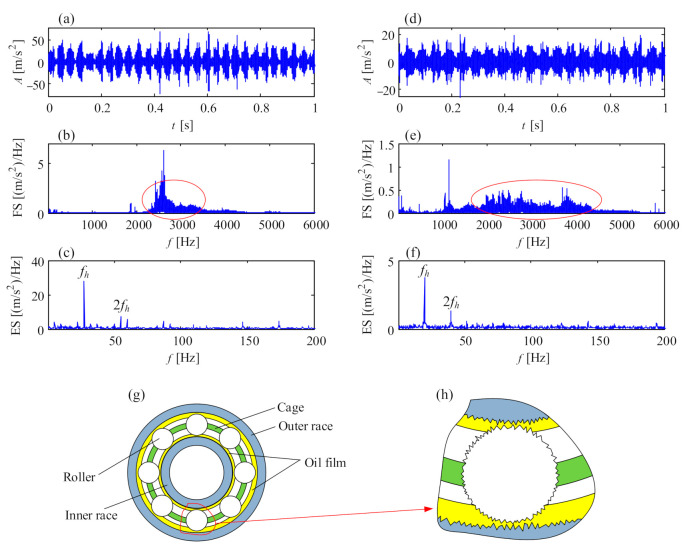
Insufficient lubrication of generator bearings: (**a**) temporal signal of bearing 1; (**b**) Fourier spectrum of bearing 1; (**c**) envelope spectrum of bearing 1; (**d**) temporal signal of bearing 2; (**e**) Fourier spectrum of bearing 2; (**f**) envelope spectrum of bearing 2; (**g**) structure of generator bearing; (**h**) friction between rolling element, inner race and outer race.

Insufficient lubrication is a key failure incentive of generator bearings. From the perspective of vibration analysis, insufficient lubrication will excite the resonance of the contact pair involving certain contact stiffness and oil stiffness. Further, the noise floor in the Fourier spectrum from 2000 to 5000 Hz is usually high, accompanied by some fault characteristic frequencies of bearing, such as rotational frequency, inner race defect frequency, outer race defect frequency or rolling element defect frequency. Figure 18 exhibits two cases of insufficient lubrication of generator bearings. The vibration signals were both from the drive end of the generator, transducer 7 in the structure of Figure 1c, with an online condition monitoring system. In the vibration waveform of Figure 18a,d, there are periodic fluctuations modulating stochastic high-frequency resonance of the contact pair. Thus, the noise floors are dominant in the Fourier spectrum of Figure 18b,e. Figure 18c,f are the envelope spectra of the enclosed frequency bands, where the rotational frequencies of generator shafts are demodulated. Compared with the acute shocks in Figure 16 of bearing looseness, the rotational frequency of the generator shaft demodulated in insufficient lubrication has less harmonics, from which it can be deduced that the eccentricity or unbalance of the generator rotor modulates the high-frequency noise of the contact pair caused by insufficient lubrication of the bearing. The noise floor is a prominent feature of insufficient lubrication of generator bearings.

Figure 18h is the zoom-in view of Figure 18g. Under insufficient lubrication, the oil film between the rolling elements and inner race or outer race could be pierced by the microcoarse surfaces; thus, the friction of the contact pair is inevitable. Since the oil stiffness of different friction positions is stochastic, the excited resonance frequency is not fixed, occupying a wide vibration frequency band, as shown in Figure 18b,e.

## 6. Challenging Issues and Solutions

Section 5 shows the fault cases with distinct fault characteristics that can be easily detected. For the diagnosis of wind turbine drivetrains, more challenging issues need to be concerned due to the complex structure of wind turbine gearboxes, varying rotational speed, potential electromagnetic interference and intensive background noise, etc. In this section, a series of challenging issues in vibration analysis for the fault detection of wind turbine drivetrains and the corresponding solutions are proposed with a perspective of published literature and our own understanding.

### 6.1. Fault Detection of Planetary Subassemblies in Wind Turbine Gearbox

**Figure 19:**
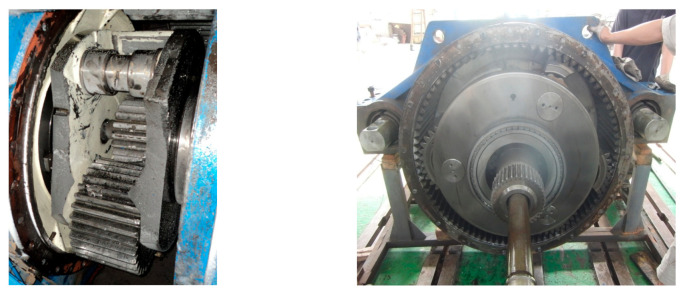
Damaged wind turbine gearbox due to planet gears failure.

Due to its compact structure and large transmission ratio, planetary gear transmission is extensively equipped at the fore end of wind turbine gearboxes. However, the adverse effects caused by planetary stage failure perform far worse than the gear failures in parallel stages. Since the planetary subassemblies, including the planet gear, sun gear and planet bearing, are enclosed by the ring gear, the debris from faulty gears or bearings always drops into the gap between the planet gear and ring gear, not falling into the oil pan like in an parallel-shaft gearbox. Further, the debris may block the revolution of the planet gear or planet carrier, further destroying the planetary subassemblies or even the whole wind turbine gearbox, which is shown in Figure 19.

Therefore, the fault diagnosis of planetary subassemblies has attracted wide research interests. In a vibration analysis of planetary transmission, the asymmetry of the modulation sidebands concerning tooth mesh frequency was explained [35], which can eliminate the confusion when observing the vibration spectrum. Further, McFadden [36] proposed a technique for calculating the time domain averages of the vibration of the individual planet gears and the sun gear in an epicyclic gearbox, in order to enhance the regular mesh vibration and counteract potential noise. Hong et al. [37] gave the explanation of frequency features in an equally spaced planetary gearbox with the aim of the fault detection of the sun gear, planet gear and ring gear. For the diagnosis of pure planetary gear sets, local mean decomposition, intrinsic time-scale decomposition and ensemble empirical mode decomposition were separately utilized to realize joint amplitude and frequency demodulation analysis for the detection of gear faults [38,39,40]. Wang et al. [41] proposed an optimal demodulation sub-band selection method for sun gear crack fault diagnosis considering the fault-related degree of each sub-band. An adaptive stochastic resonance method [42] and its combination with ensemble empirical mode decomposition [43] were adopted to extract weak fault characteristics from noisy signals. Autocorrelation-based time synchronous averaging (ATSA) was proposed to solve the signal distortion of conventional TSA and applied for the fault diagnosis of planet gears [44]. Liang et al. [45] developed a windowing and mapping strategy to find the weak fault symptom generated by a single cracked tooth in a planet gear. Feng and Liang [46] utilized the shift invariant K-means singular value decomposition dictionary learning method to suppress background noise and reveal vibration patterns of planetary gearbox vibration signals.

In a wind turbine gearbox with both planetary and parallel stages, in addition to the varying transmission path caused by the revolution of the planet carrier, the fault characteristics of planetary subassemblies can be hidden by the mesh energy of the intermediate stage, high speed stage or background noise. On a scale-down testbed of a wind turbine gearbox, Wang et al. [47,48] designed MRgram to find the meshing resonance frequency band in vibration signals and demodulated the fault characteristic of the ring gear. When distributed faults emerged on the planetary gears, a novel modulation phenomenon was demonstrated in [49], which was described as the mesh frequency of the intermediate stage, high speed stage or mechanical natural frequency of the gearbox was a carrier wave modulated by the mesh frequency of planetary stage. For the local fault detection of planetary gears in a wind turbine gearbox, Feng and Liang [50] applied iterative atomic decomposition thresholding to enhance the gear characteristic frequencies of interest, and verified its effectiveness in the fault detection of the planet gear of an on-site wind turbine gearbox. Spectral kurtosis was recognized as a powerful tool for the detection of a tooth crack in the ring gear of a wind turbine gearbox [51].

Planet bearings are another key part in planetary gear transmission, which support planet gears for autorotation. The fault characteristic of planet bearing is weaker than that of the bearing supporting fixed-axis gear or rotor because of the long and varying transmission path from fault source to accelerometer. To solve the particularly difficult diagnostic problem of planet bearing, Bonnardot et al. [52] proposed a combination of unsupervised noise cancellation and angular resampling to suppress noise and speed fluctuation. Fan and Li [53] pointed out that the internal vibration transducer performed better than the traditional transducer on the casing in detecting planet bearing fault. Unfortunately, the current wind turbine gearbox cannot provide locations for internal vibration transducers. Wang et al. developed SKRgram [54] and mesh frequency modulation index-based demodulation techniques [55] to find the sensitive frequency band hiding fault characteristic of the planet bearing. Feng et al. [56] found that the informative frequency band (including the center frequency and bandwidth) of the planet bearing fault induced repetitive impulses using the spectral negentropy-based infogram. The importance of the planet bearing was investigated in [57], and the vibration signatures of planet bearing inner- and outer-race defects were determined by a dynamic model of a wind turbine planetary drivetrain.

**Figure 20:**
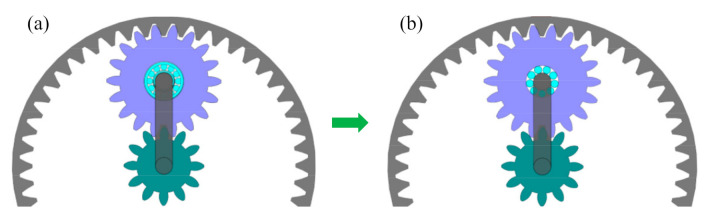
Comparison of planet bearings: (**a**) original bearing; (**b**) improved bearing.

Another common failure of planet bearings in wind turbine gearboxes is looseness, i.e., the looseness between the inner race and planet carrier, or between the outer race and planet gear. Looseness of planet bearings will lead to abrasive wear and unsteady support of the planet gear, causing destructive results for the wind turbine gearbox. In order to avoid the looseness of planet bearings, the structure of the planet bearing is improved from Figure 20a,b without inner race and outer race.

The following are two naturally damaged fault cases of planetary subassemblies in a wind turbine gearbox.

#### 6.1.1. Local Fault of Planet Gear

**Figure 21:**
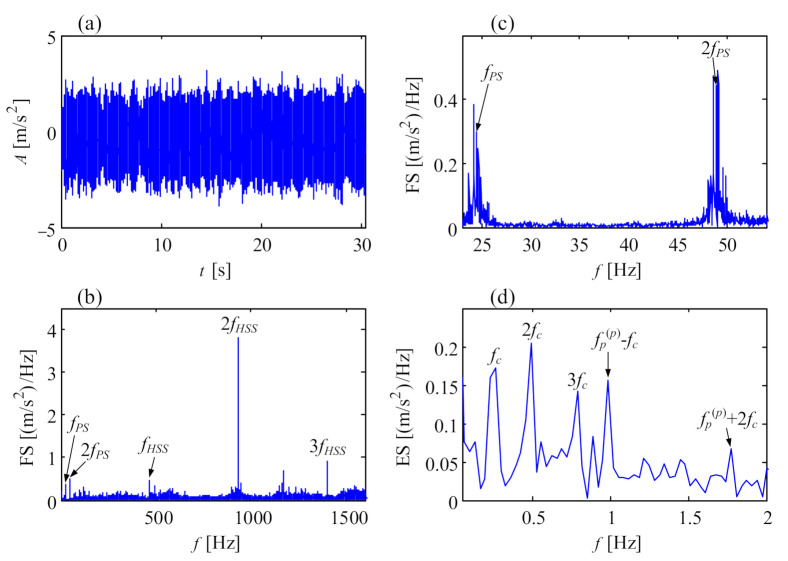
Vibration signal of chipped teeth of planet gear: (**a**) temporal signal; (**b**) Fourier spectrum; (**c**) Fourier spectrum near the mesh frequency of planetary stage; (**d**) envelope spectrum.

**Figure 22:**
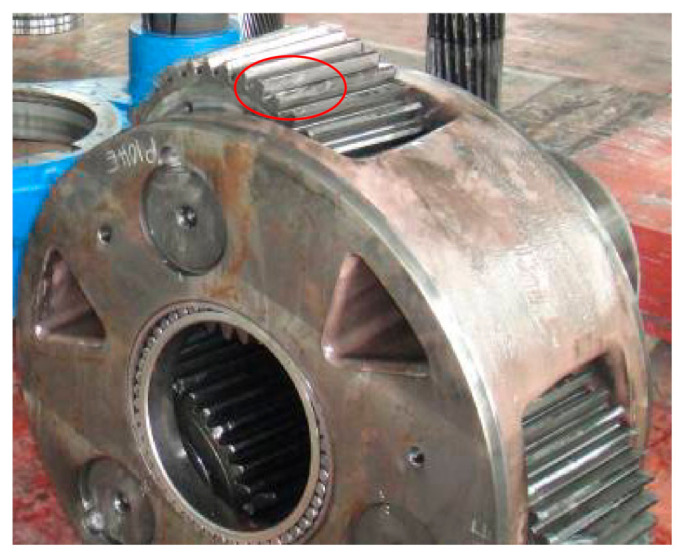
Chipped teeth of planet gear in a wind turbine gearbox.

Figure 21a shows the vibration signal from a 750 kW wind turbine gearbox with chipped planet gears, as shown in Figure 22. The vibration amplitudes are low, within ±4 m/s^2^. In the Fourier spectrum of Figure 21b, the mesh frequency of the high-speed stage and its harmonics are the dominant frequencies, and the mesh frequency of the planetary stage takes a back seat. Figure 21c is the zoom-in view of Figure 21b between 23 and 54 Hz, where some sidebands stand around the mesh frequency of planetary stage. By demodulating the frequency band between 23 and 30 Hz, the envelope spectrum can be shown in Figure 21d where the rotational frequency *f_c_* of the planet carrier and its harmonics is distinct. Simultaneously, the fault frequencies $f_{p}^{\left(p\right)}-f_{c}$ and $f_{p}^{\left(p\right)}+2f_{c}$ indicating local fault of the planet gear appear clearly, which matches the chipped teeth in Figure 22. Here $f_{p}^{\left(p\right)}=2f_{PS}/Z_{p}$. From this case, we can see that although chipped teeth fault emerges on the planet gear, its fault characteristic frequency is so weak even if the frequency bands near the mesh frequency of the planetary stage are considered.

#### 6.1.2. Distributed Fault of Planetary Stage

**Figure 23:**
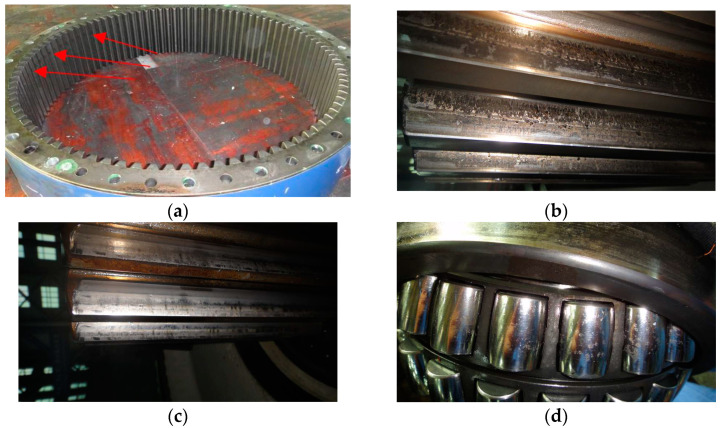
Distributed fault of planetary subassemblies in an 850 kW wind turbine gearbox: (**a**) ring gear; (**b**) pitting planet gear; (**c**) deformed sun gear; (**d**) pitting rolling elements of planet bearing.

A distributed fault case of planetary stage in a wind turbine gearbox is shown in Figure 23. There are weak indentations on the ring gear and severe pitting on the planet gear. The teeth surfaces of the sun gear are squashed, and pitting emerges on the rolling elements of the planet bearing.

**Figure 24:**
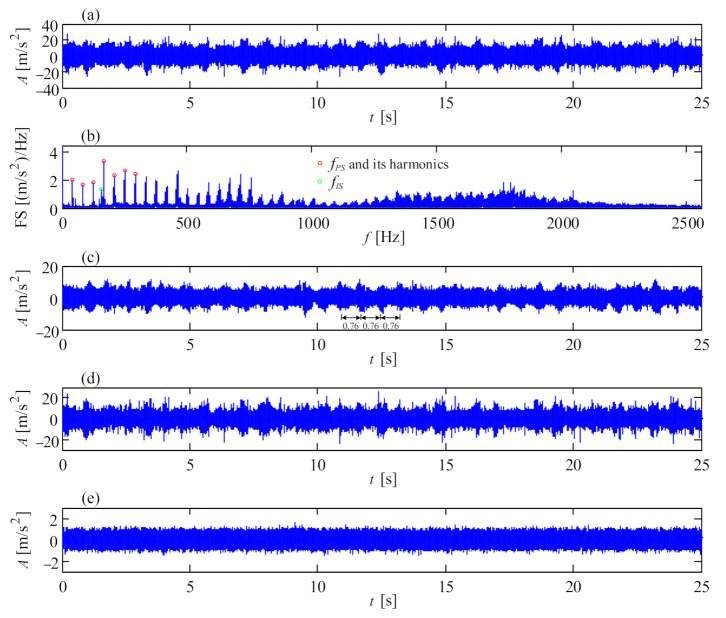
Demodulation results by resonance-based sparse decomposition: (**a**) vibration signal from faulty planetary subassemblies; (**b**) Fourier spectrum; (**c**) high Q component; (**d**) low Q component; (**e**) noise component.

The vibration signal from faulty planetary subassemblies is shown in Figure 24a where the stochastic component and shock component mix together. The Fourier spectrum of the vibration signal is shown in Figure 24b. The mesh frequency *f_PS_* of the planetary stage and its harmonics occupy almost all of the spectrum from low frequency to high frequency, accompanied by the weaker mesh frequency *f_IS_* of the intermediate stage. The phenomenon can be interpreted as: the worn or deformed surfaces of planetary subassemblies change the meshing stiffness of the planetary stage, which enhances the vibration energy during the planet gear meshing with the sun gear and ring gear. In addition to the *f_PS_* and *f_IS_*, other fault information cannot be detected. Then, the signal in Figure 24a is decomposed into a high quality factor (high Q) component, low Q component and noise, by referring to the tunable Q-factor wavelet transform [58], and resonance-based sparse decomposition [59]. In the high Q component of Figure 24c, there are regular fluctuations with intervals of 0.76 s (1.315 Hz), denoting three times the rotational frequency of the planet carrier. This interval in the high Q component indicates potential distributed defects on the ring gear.

**Figure 25:**
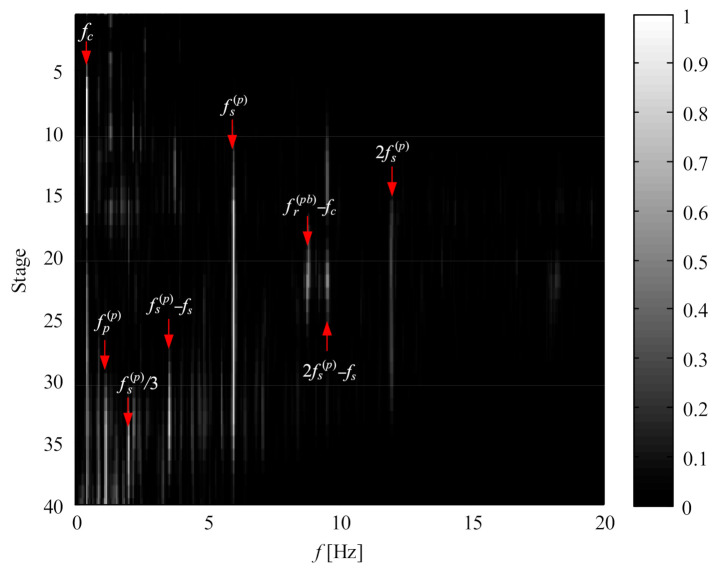
Normalized multistage enveloping spectrogram of low component.

Gear or bearing faults generally exhibit low Q-factor properties; thus, the low Q component in Figure 24d is decomposed into a series of wavelet coefficients which are further re-constructed into the corresponding sub-band signals. The normalized enveloping spectrogram of these sub-band signals is shown in Figure 25 where the fault characteristic frequencies $f_{s}^{\left(p\right)}$, $2f_{s}^{\left(p\right)}$, $f_{s}^{\left(p\right)}/3$, $\left(f_{s}^{\left(p\right)}-f_{s}\right)$ and $\left(2f_{s}^{\left(p\right)}-f_{s}\right)$ denoting the sun gear fault are detected. $\left(f_{r}^{\left(pb\right)}-f_{c}\right)$ denoting the rolling element fault of planet bearing appears, and the frequency $f_{p}^{\left(p\right)}$ denoting the planet gear fault is found as well. Here, $f_{p}^{\left(p\right)}=f_{PS}/Z_{p}$. All the fault characteristic frequencies are in accordance with the faulty subassemblies in Figure 23. This case [60] demonstrates that the signal separation technique is effective in detecting the fault information of planetary subassemblies hidden in complex noise.

From the analysis of the research above, spectral kurtosis and its variants are beneficial to extract the local fault features of planetary gears in real wind turbine gearboxes with multistage gear transmission. Resonance-based sparse decomposition is effective in excluding the interference from the meshing vibration caused by the intermediate stage or high-speed stage, so that the distributed fault of planetary subassemblies can be extracted.

### 6.2. Fault Feature Extraction under Nonstationary Conditions

Wind turbines operate under nonstationary conditions due to varying stochastic wind speeds; thus, time-frequency analysis for vibration signals from wind turbine drivetrains is attractive. In [61], some time-frequency algorithms that could be used for the analysis of vibration signals of wind turbine drivetrains were reviewed. Time-frequency representation and order analysis are primary methods for the fault detection of wind turbine drivetrains under nonstationary conditions.

For the vibration signal of a wind turbine gearbox, multicomponent modulation sidebands indicating gears or bearings defect are common, which causes some restrictions to wind turbines while applying conventional time-frequency representation. A general framework for parameterized time-frequency transforms was presented by Yang et al. [62]. Under this framework, when spline-kernel chirplet transform (SCT) was selected, it was more accurate than other methods for estimating the varying instantaneous frequency of mono-component signals [63]. Considering the multicomponent property of wind turbine vibration signals, an improved SCT was developed in [64] with a view to extract the instantaneous amplitude of a multicomponent signal at fault-related frequencies of interest. Guan et al. [65] used generalized demodulation to demodulate a signal into several signals based on the shaft rotational speed to meet the constant-frequency requirement of bilinear distribution, and applied Cohen’s class bilinear distribution to obtain the time-frequency representation of the demodulated signals. This method was effective in planetary gearboxes under time-varying speed and fixed external load conditions. The original vibration signal of a planetary gearbox could be decomposed into multiple mono-components by iterative generalized demodulation [66], and was realized by time-frequency demodulation analysis. Synchrosqueezing transform was improved using iterative generalized demodulation [67] to identify multicomponent and time-variant frequency signals from a planetary gearbox under nonstationary conditions. Optimal kernel time-frequency analysis [68], Vold-Kalman filter and higher order energy separation [69] and iterative generalized time-frequency reassignment [70] were developed in succession by Feng’s team to exhibit the time-frequency representation of vibration signals from a faulty planetary gearbox under nonstationary conditions. A positive energy residual method based on wavelet transform [71] and Gaussian process was proposed by Park et al. [72] to remove the variability of signals while detecting faults of a planetary gear under variable speed conditions. An iterative generalized demodulation-based order spectrum analysis method was exploited to convert arbitrary instantaneous frequency trajectories of multicomponent signals into constant frequency lines [73], which can effectively reveal the harmonic order constituents of nonstationary multicomponent signals from faulty planetary gearboxes. A tacholess tracking method termed dual path optimization ridge estimation was proposed to detect planet bearing defects in a planetary gearbox under varying speed operation [74]. Hong et al. [75] proposed a tacholess diagnostic technique based on the optimal warping path evaluated from the fast dynamic time-warping algorithm, which can identify fault information of the sun gear and annulus gear in a wind turbine gearbox under speed fluctuations.

For the parts except planetary gears in a wind turbine gearbox, nonstationary analysis methods were addressed as well. A discrete spectrum correction technique-based order tracking method was proposed by He et al. [76] to find the misalignment of the output shaft in a wind turbine gearbox from the long nonstationary vibration signal. Li et al. [77] incorporated variational mode decomposition into convolutive blind-source separation to address the challenge of substantial driving-speed variations in bearing fault detection in a wind turbine gearbox. Antoniadou et al. [78] found gear faults in the intermediate stage and high-speed stage under varying load conditions in a wind turbine gearbox using empirical mode decomposition and a Teager–Kaiser energy operator. A gear parameter identification method was proposed in [79] to determine the numbers of teeth in a wind turbine gearbox with one planetary and two helical parallel stages under varying speed, which can be used for carrying out vibration-based fault diagnosis. A review on the angular resampling algorithm for application in conditions of high-speed variability in wind turbines was presented to develop a wind turbine diagnostic system [80]. In direct-drive wind turbines, the bearings with low rotational speed are subject to varying operational conditions; Pezzani et al. [81] proposed a resampling technique that determined the rotor position by the phase-locked loop synchronized generator voltages, which was applied for the vibration-based fault detection of a bearing in a permanent magnet synchronous generator.

It is noteworthy that during a vibration test within a relatively short duration (e.g., over 16 s for main bearings and planetary stage; 4 s for intermediate stage, high-speed stage and generator bearings), wind turbines keep an approximately constant rotational speed due to the huge inertia of rotor system with heavy blades. Comparatively speaking, nonstationary signal processing is more suitable for the vibration analysis of main bearings and planetary gears with lower rotational speed, because the rotational speeds of these parts are prone to variation during longer sampling durations. Apparently, the time-frequency analysis of multicomponent with high time-frequency concentration is a promising tool to diagnose low-speed mechanical parts.

### 6.3. Fault Information Enhancement of Vibration Signal

Harsh operational environment and complex structures cause numerous disturbances to the measured vibration signal collected from wind turbine drivetrains. Techniques of fault information enhancement need to be developed to detect weak fault features masked in intensive interferences or noise.

#### 6.3.1. Compound Faults in Wind Turbine Gearbox

**Figure 26:**
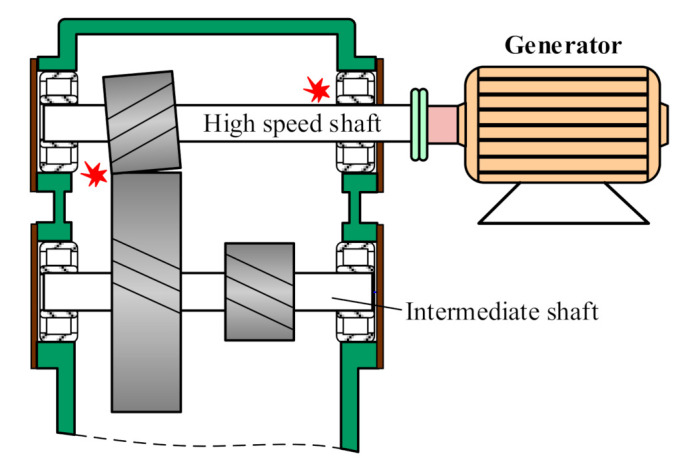
Failure mechanism of compound faults in a wind turbine gearbox.

**Figure 27:**
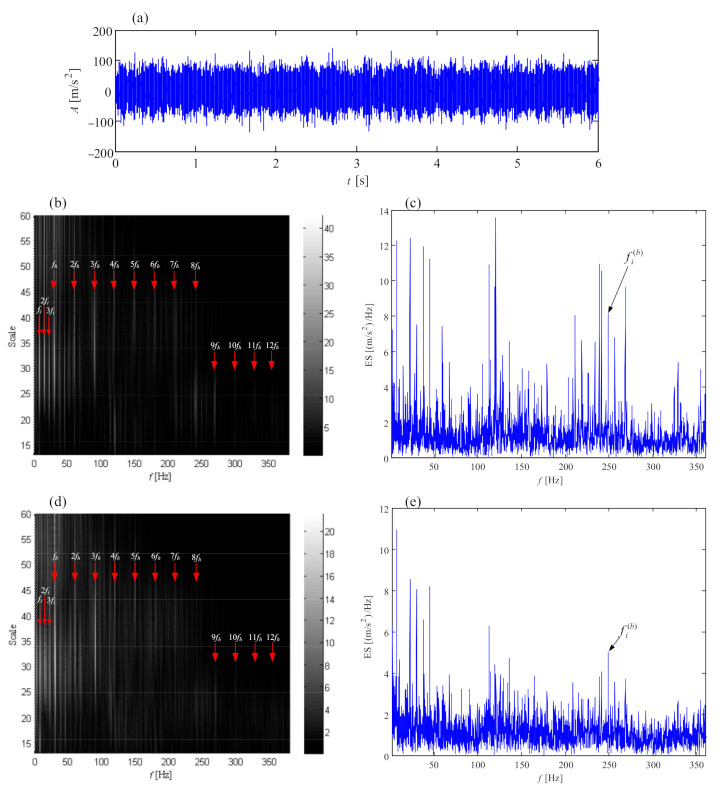
Multiscale enveloping spectrogram: (**a**) vibration signal from faulty gear pair and rear bearing; (**b**) MuSEnS before cepstrum pre-whitening; (**c**) slice of the MuSEnS at scale 20; (**d**) MuSEnS after cepstrum pre-whitening; (**e**) slice of the MuSEnS at scale 20.

Wind turbine gearbox consists of multiple gears and bearings, so that compound faults are extremely frequent due to the dynamic coupling among diverse parts. Faulty bearing may lead to insufficient stiffness to support the shaft with gears so that the shaft may tilt. The torque transmitted by the whole face width of the meshed gear pair is now transmitted by partial face width; thus, the gear pair is prone to breakage. A failure case [82] including rear bearing and a gear pair of the high-speed stage in a wind turbine gearbox is shown in Figure 26. In this type of compound fault, gear faults commonly cause intensive vibration, easily hiding the fault feature of bearing. The vibration signal from the failure case is shown in Figure 27a; complex Gaussian wavelet transform [71] is utilized to decompose the vibration signal at different scales. The multiscale enveloping spectrogram (MuSEnS) [83] after complex wavelet transform is shown in Figure 27b, where *f_i_* denoting the faulty wheel on the intermediate shaft and *f_h_* denoting the faulty pinion on the high-speed shaft are clear. In the slice at scale 20 in Figure 27c, the fault frequency $f_{i}^{\left(b\right)}$ denoting the inner race fault of the rear bearing on the high-speed shaft emerges but is covered by the rotational frequencies of the intermediate shaft and high-speed shaft denoting the gear fault. After the cepstrum pre-whiting analysis [84] for the original signal, the MuSEnS is shown in Figure 27d. From the slice at the same scale, the $f_{i}^{\left(b\right)}$ is dominant among its neighboring frequency components in Figure 27e because of the suppression effect of cepstrum pre-whiting for the meshing vibration of the faulty gear pair.

In the situation of multiple gear failure, the fault information of the defective gear with a lower rotational frequency is generally concealed by the one with a higher rotational frequency. MuSEnS is still effective in exhibiting the fault characteristic frequencies of the cracked gear on the sun shaft and the broken gear on the intermediate shaft, at different scales [85]. Sparse feature identification based on union of redundant dictionary [86] was presented to separate the fault information of the gear on the intermediate shaft from the fault feature of misalignment of the high-speed shaft in a wind turbine gearbox.

#### 6.3.2. Signal Decomposition Methods

Signal decomposition techniques enable the decomposition of the vibration signal into a series of sub-band signals, automatically locating fault frequency bands and manifesting fault information from potential interferences or noise. Teng et al. [87] applied the classic empirical mode decomposition to find the fault characteristics of pitting in the gear pair of the high-speed stage in a wind turbine gearbox. Liu et al. [88] used the local mean decomposition (LMD) to decompose the vibration signal into a set of functions, each of which was the product of an envelope signal and a frequency modulated signal. The instantaneous frequency from the frequency modulated signal was identified as an indicator of crack faults of gear on the high-speed shaft. To suppress the end effect of LMD, an integral extension LMD based on the integral local waveform matching of the right and left side of the original signal [89] was presented for wind turbine ball bearing fault diagnosis. Wang et al. [90] utilized ensemble empirical mode decomposition to decompose one-channel vibration measurements into a series of intrinsic mode functions as pseudo-multichannel signals, and performed independent component analysis on the intrinsic mode function to separate bearing defect-related signals from gear meshing signals in a wind turbine gearbox. Hu et al. [91] applied ensemble intrinsic time-scale decomposition, wavelet packet decomposition and correlation dimension to identify different fault types of wind turbine gearbox. As a recently proposed signal processing method, empirical wavelet transform was used to extract inherent modulation information by decomposing the signal into mono-components and identifying fault features of generator bearing in a wind turbine [92]. A parameterless empirical wavelet transform without any prior knowledge was introduced and applied for the multifault extraction of generator bearing in a wind turbine [93].

#### 6.3.3. Bearing Fault in Wind Turbine Generator under Intensive Interference

**Figure 28:**
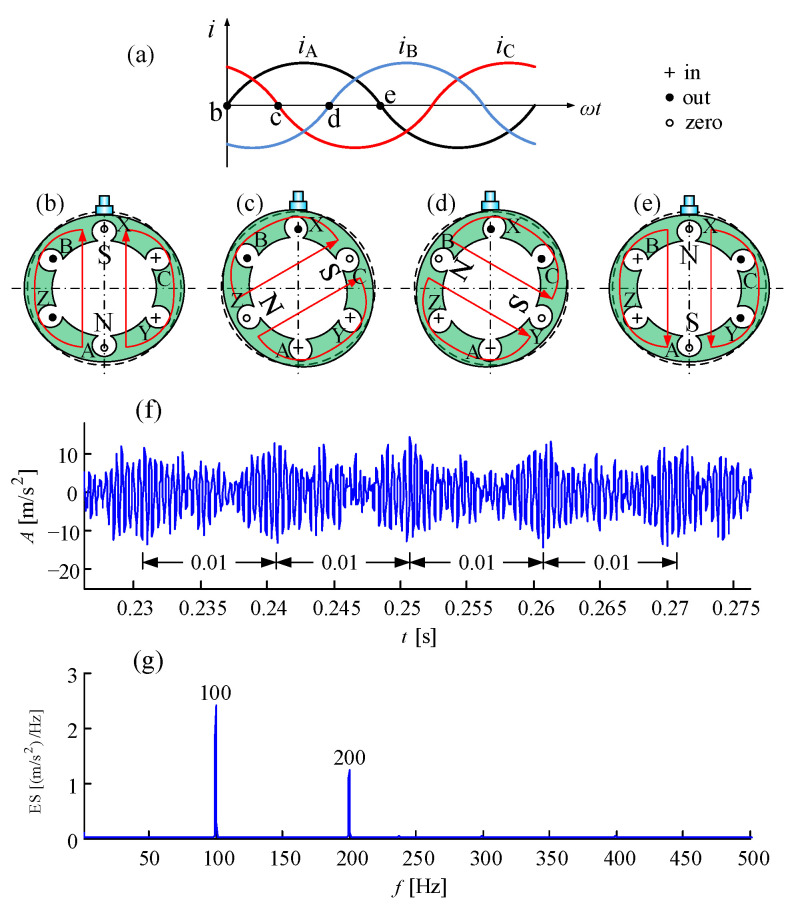
Electromagnetic vibration of generator stator: (**a**) three phase alternating currents; (**b**) deformation at time b; (**c**) deformation at time c; (**d**) deformation at time d; (**e**) deformation at time e; (**f**) modulated vibration signal; (**g**) demodulated electromagnetic vibration.

Electromagnetic vibration is a common phenomenon acting on the hollow stator of a wind turbine generator, because of the structure with low weight and stiffness in high altitude. The principle of electromagnetic vibration is shown in Figure 28 [94]. With the input of three phase currents in Figure 28a, a rotational magnetic field emerges at a synchronous speed of current frequency. The two poles of the magnetic field attract each other, which can cause stator deformation. During the half period of alternating current from b to e in Figure 28a, the deformation varies, shown in Figure 28b–e. These deformations have a modulation effect on the vibration signal shown in Figure 28f, and the demodulated frequency of 100 Hz and its harmonic are distinct in Figure 28g.

**Figure 29:**
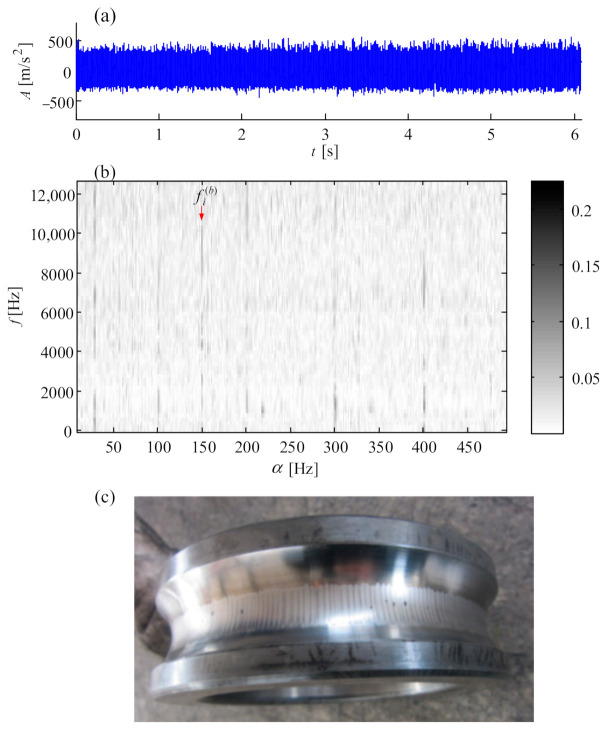
Vibration analysis of a faulty bearing signal: (**a**) faulty vibration signal polluted by electromagnetic vibration; (**b**) cyclic coherence function of the vibration signal; (**c**) faulty inner race of the bearing.

A vibration signal from a faulty bearing at the drive end of a wind turbine generator is shown in Figure 29a. The signal is polluted by the electromagnetic vibration. The cyclic coherence function [95], which can enhance the amplitudes at cyclic frequency denoting fault frequency and restrain those at cyclic frequency denoting noise, was utilized to extract the fault characteristic of bearing [94]. The cyclic coherence function of the vibration signal in Figure 29a is shown in Figure 29b where the fault characteristic frequency $f_{i}^{\left(b\right)}$ of the inner race is obvious among the electromagnetic vibration of 100 Hz and its harmonics. The faulty inner race of the bearing is shown in Figure 29c. The cyclic coherence function was also adopted for the diagnosis of compound fault features under strong rotor imbalance, including the inner race and outer race of the bearing in the wind turbine generator [96].

#### 6.3.4. Denoising for Fault Enhancement

Noise is not to be neglected in wind turbine drivetrains, which may interfere with the extraction of fault characteristics. Sun et al. [97] applied a multiwavelet denoising technique with a data-driven block threshold for the fault detection of the rolling bearing with a slight inner race defect in a wind turbine generator. The usage of the minimum entropy deconvolution technique has shown a strong enhancement in the fault detection of an inner race fault in wind turbine generators [98]. The Morlet wavelet [71], whose shape was similar to mechanical fault signals, was chosen as a denoising tool for the raw vibration signals from a wind turbine gearbox [99,100]. A denoising and feature extraction method was developed [101] by adding empirical mode decomposition and autocorrelation denoising to the wavelet package transform under the effects of white noise and short-term disturbance noise in wind turbine vibration signals. Li et al. [102] presented a new noise-controlled second-order enhanced stochastic resonance method based on the Morlet wavelet transform to extract fault features for wind turbine vibration signals. A frequency-shift multiscale noise tuning stochastic resonance method [103] was proposed to enhance weak signal features in the fault diagnosis of generator bearing in wind turbines. A cross genetic algorithm optimal Mexican-hat wavelet [71] was proposed, which can be used for weak feature extraction from the strong background noise in a wind turbine gearbox [104]. Hong and Dhupia [105] developed a new time-domain fault detection method combining fast dynamic time wrapping as well as correlated kurtosis to characterize the local gear fault in a planetary gearbox, which is beneficial in practical analysis to highlight sideband patterns in situations where data are often contaminated by process/measurement noises. Li et al. [106] presented a new method based on the supervised order tracking bounded component analysis for gear crack detection in wind turbines, which incorporated order tracking to eliminate noise and disturbance signal components.

In summary, fault information enhancement plays a significant role in the fault detection of wind turbine drivetrains. Multiscale envelope, signal decomposition, cyclic coherence function and signal denoising are the critical solutions while encountering the vibration signal with multipart faults under intensive background noise.

### 6.4. Health Indicator for Vibration-Based Condition Monitoring

Vibration-based condition monitoring not only helps maintainers to diagnose the mechanical components of wind turbines but provides health indicators which can assist remaining life prediction. Shanbr et al. pointed out that energy index was a superior indicator, representing the crack severity and progress of wind turbine bearing [107], which was defined as the square of the ratio of the root mean square of a defined segment in a given signal to the overall root mean square of the same signal. A gear condition indicator, the sideband power factor [108], was proposed to evaluate the gear damage in the high-speed stage of a wind turbine during nonstationary load and speed operating conditions. Pattabiraman et al. [109] presented a sideband energy ratio for the monitoring of gear defect progression in wind turbine gearboxes. Ni et al. [110] showed the advantages of sample entropy features in detecting and evaluating the progress of early faults of the rolling bearing in a wind turbine transmission system. Guo et al. [111] utilized correlation and monotonicity to select sensitive features from original classical features, including mean, RMS and kurtosis, and further constructed a recurrent neural network-based health indicator for wind turbine generator bearings.

**Figure 30:**
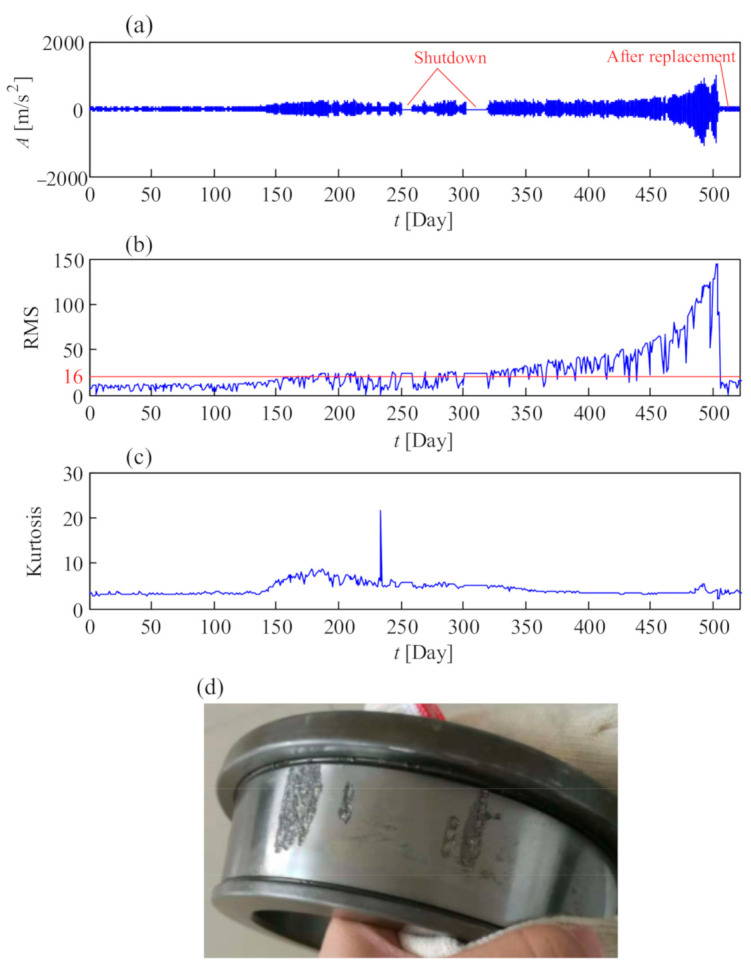
A life cycle vibration case from faulty rear bearing on the high-speed shaft of wind turbine gearbox: (**a**) temporal signal; (**b**) RMS; (**c**) kurtosis; (**d**) defective inner race of the bearing.

Figure 30a shows a life cycle vibration signal from the faulty rear bearing on the high-speed shaft in a 2 MW wind turbine gearbox. The signal is listed day by day. Before the 150th day, the bearing had a healthy status and the vibration amplitude was low. The root mean square (RMS), as shown in Figure 30b, was lower than the alarm threshold in VDI 3834 [33] during that period. After the 150th day, the RMS fluctuated around the alarm threshold until the 350th day; meanwhile, the kurtosis in Figure 30c between the 150th and 350th day performed better than that during the first 150 days, indicating that the incipient fault emerged on the bearing. The peak value in the kurtosis of Figure 30c indicated potential spalling from the bearing at that time. Sometimes, the RMS fell below the threshold, which was caused by the low rotational speed during the vibration test. After the 350th day, the vibration amplitude and RMS increased gradually, indicating further deterioration of the bearing health condition until the bearing was replaced on the 500th day. The failure bearing is shown in Figure 30d. After replacement of the bearing, the vibration amplitude fell below the threshold. From this case, we can see that RMS can be treated as a reasonable indicator for the remaining useful life prediction of the bearing on the high-speed shaft of the wind turbine gearbox.

### 6.5. Pedestal Looseness

**Figure 31:**
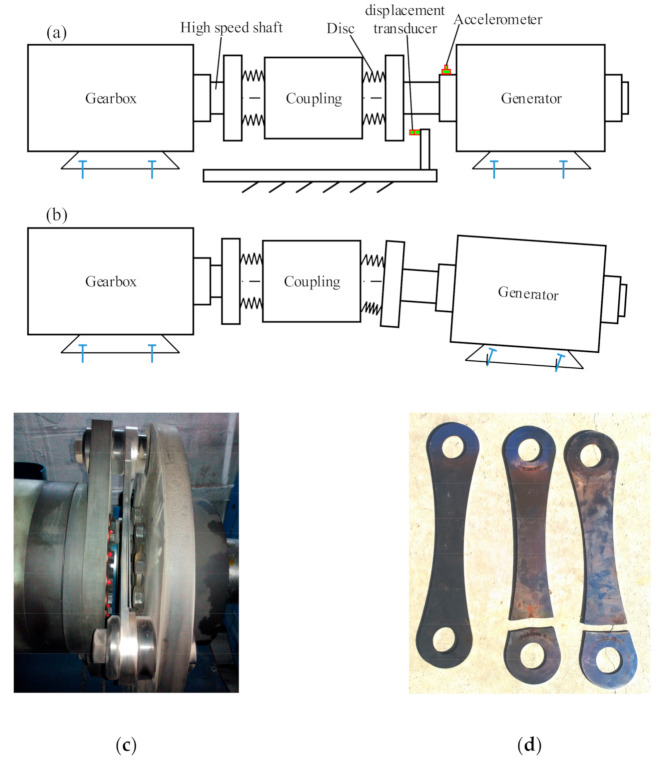
Disc coupling between the high-speed shaft of wind turbine gearbox and generator shaft: (**a**) disc coupling under normal state; (**b**) disc coupling under pedestal looseness; (**c**) broken discs in coupling; (**d**) disassembled discs.

Disc coupling is widely utilized to connect the high-speed shaft of the wind turbine gearbox with the generator shaft, which is shown in Figure 31. The misalignments of the two shafts are compensated by the deformation of the discs in order to protect the connected shafts and bearings. However, pedestal looseness, such as looseness of the foundation bolt of the generator, may enhance the relative displacements of the connected two shafts, causing destructive stress to the discs, as shown in Figure 31b. A broken coupling and discs in a wind turbine drivetrain are shown in Figure 31c,d, which are caused by the pedestal looseness of the generator. For the detection of pedestal looseness, the noise-controlled second-order enhanced stochastic resonance method [102] was reported by means of twice integral transform for amplifying weak fault information on the noisy signal. An et al. [112] used a displacement transducer to acquire the vibration signal of a direct-drive wind turbine and investigated ensemble empirical mode decomposition and Hilbert transform [32] to extract the feature of bearing pedestal looseness. Compared with the acceleration-based method, the displacement-based method is more applicable in pedestal looseness detection, because the real misalignment force can be absorbed by the deformation of the discs and hardly be reflected by the accelerometer mounted on the surface of the generator. Figure 31a shows the displacement transducer which is used to measure the axial displacement of the flange of the coupling, which can directly reflect the pedestal looseness of the generator.

## 7. Research Needs and Future Challenges

To date, numerous organizations and companies exist that provide commercial vibration analysis for wind turbine drivetrains. In summary, several challenges need to be faced to prompt the prosperous development of wind energy, which are listed as follows:

(1) The parameters of the vibration data acquisition system, e.g., the sensitivity of accelerometer, measurement range and frequency response range, are various, accounting for nonstandard data sources from wind turbine drivetrains. Urgent work is expected to standardize the condition monitoring system for manufacturers, researchers and operators.
(2) During the operation of wind turbines, massive vibration signals are generated each second, which causes a challenge for data archiving and communication. It is suggested that the instantaneous vibration signal should be stored once four hours, with a 30 s duration each time. The signals at other times are processed as status parameters in order to save storage space.
(3) The fault detection for planetary subassemblies in wind turbine gearboxes is still an intractable task due to varying transmission path and adjacent fault characteristic frequencies caused by low rotational speed. The higher vibration energy from intermediate and high-speed stages also masks the fault features of planetary subassemblies.
(4) After extracting the fault characteristics and constructing the health indicator, remaining useful life prediction is a significant task that can help schedule the maintenance of gearboxes or generators for wind turbines.
(5) Vibration analysis and fault diagnosis for wind turbine drivetrains mainly depend on the experiences of professional engineers, which lacks intelligent diagnosis function. Certainly, it is necessary to take account of the following situations in the intelligent diagnosis of wind turbine drivetrains: different sampling frequency at different measurement positions, complex drivetrain structure, varying working condition, etc.

## 8. Conclusions

This paper reviews the research on the fault detection of wind turbine drivetrains in the past decade. The structures of the mainstream wind turbine drivetrains are introduced, and the computation of fault characteristic frequencies of the mechanical components are given. Five common mechanical faults in wind turbine drivetrains are proposed with their signal features being interpreted. For the challenging issues, signal separation techniques, e.g., sparse decomposition, are significant for the fault detection of planetary subassemblies affected by the vibration from intermediate and high-speed stages; the time-frequency analysis of multi-component, high time-frequency concentration should be implemented for the fault feature extraction of low speed parts under nonstationary conditions, e.g., planetary gears and main bearings; multiscale envelope, signal decomposition and signal denoising need to be pertinently studied to exhibit weak faults hidden in strong interferences and noise; the construction of a health indicator is pivotal in remaining useful life prediction; displacement-based measurement is recommended to detect pedestal looseness online.

In the future, wind energy will maintain a rapid development trend in China due to the policy of renewable energy. The fault detection of wind turbine drivetrains is expected to be more accurate, automatic and intelligent, which will help to reduce the labor under harsh environments and the operational costs at wind farms.

## Figures and Tables

**Table 1:** Mesh frequencies of different stages in the gearbox with one planetary stage and two parallel stages.

	Formula
Planetary stage	$f_{PS}=f_{c}\cdot Z_{r}=(f_{s}-f_{c})Z_{s}$
Intermediate stage	$f_{IS}=f_{s}Z_{mi}=f_{i}Z_{mo}$
High speed stage	$f_{HSS}=f_{i}Z_{hi}=f_{h}Z_{ho}$

**Table 2:** Rotational frequencies of different shafts in the gearbox with one planetary stage and two parallel stages.

	Formula
Sun shaft	$f_{s}=f_{c}\left(1+Z_{r}/Z_{s}\right)$
Intermediate shaft	$f_{i}=f_{s}\cdot Z_{mi}/Z_{mo}$
High-speed shaft	$f_{h}=f_{i}\cdot Z_{hi}/Z_{ho}$

**Table 3:** Mesh frequencies of different stages in the gearbox with two planetary stages and one parallel stage.

	Formula
The first planetary stage	$f_{PS1}=f_{c1}\cdot Z_{r1}=(f_{c2}-f_{c1})Z_{s1}$
The second planetary stage	$f_{PS2}=f_{c2}\cdot Z_{r2}=(f_{s2}-f_{c2})Z_{s2}$
High speed stage	$f_{HSS}=f_{s2}Z_{hi}=f_{h}Z_{ho}$

**Table 4:** Rotational frequencies of different shafts in the gearbox with two planetary stages and one parallel stage.

	Formula
The sun shaft of the first PS (The planet carrier of the second PS)	$f_{c2}=f_{c1}\left(1+Z_{r1}/Z_{s1}\right)$
The sun shaft of the second PS	$f_{s2}=f_{c2}\left(1+Z_{r2}/Z_{s2}\right)$
High-speed shaft	$f_{h}=f_{s2}\cdot Z_{hi}/Z_{ho}$

**Table 5:** Fault characteristic frequency of planetary gear transmission.

	Only Faulty Gear Considered	Potential Combination
Planet gear	$f_{p}^{\left(p\right)}=f_{PS}/Z_{p}$	$k\cdot f_{p}^{\left(p\right)}\pm n\cdot f_{c}$
Sun gear	$f_{s}^{\left(p\right)}=3f_{PS}/Z_{s}$	$k\cdot f_{s}^{\left(p\right)}\pm n\cdot f_{s}$
Ring gear	$f_{r}^{\left(p\right)}=3f_{PS}/Z_{r}=3f_{r}$	

**Table 6:** Fault characteristic frequency of bearing supporting fixed-axis gear or rotor.

	Formula
Inner race	$f_{i}^{\left(b\right)}=\frac{f_{r}N_{b}}{2}\left(1+\frac{d}{D}\cos\varphi \right)$
Outer race	$f_{o}^{\left(b\right)}=\frac{f_{r}N_{b}}{2}\left(1-\frac{d}{D}\cos\varphi \right)$
Rolling elements	$f_{r}^{\left(b\right)}=\frac{f_{r}D}{2d}\left(1-{\left(\frac{d}{D}\cos\varphi \right)}^{2}\right)$
Cage	$f_{c}^{\left(b\right)}=\frac{f_{r}}{2}\left(1-\frac{d}{D}\cos\varphi \right)$

**Table 7:** Fault characteristic frequency of planet bearing.

	Only Faulty Bearing Parts Considered	Potential Combination
Inner race	$f_{i}^{\left(pb\right)}=\frac{f_{p}^{\left(p\right)}N_{b}}{2}\left(1+\frac{d}{D}\cos\varphi \right)$	$k\cdot f_{i}^{\left(pb\right)}\pm n\cdot f_{c}$
Outer race	$f_{o}^{\left(pb\right)}=\frac{f_{p}^{\left(p\right)}N_{b}}{2}\left(1-\frac{d}{D}\cos\varphi \right)$	$k\cdot f_{o}^{\left(pb\right)}\pm m\cdot f_{p}^{\left(p\right)}\pm n\cdot f_{c}$
Rolling elements	$f_{r}^{\left(pb\right)}=\frac{f_{p}^{\left(p\right)}D}{2d}\left(1-{\left(\frac{d}{D}\cos\varphi \right)}^{2}\right)$	$k\cdot f_{r}^{\left(pb\right)}\pm m\cdot f_{c}^{\left(pb\right)}\pm n\cdot f_{c}$
Cage	$f_{c}^{\left(pb\right)}=\frac{f_{p}^{\left(p\right)}}{2}\left(1+\frac{d}{D}\cos\varphi \right)$	$k\cdot f_{c}^{\left(pb\right)}\pm n\cdot f_{c}$

**Table 8:** Layout of accelerometers of vibration monitoring system.

No.	Frequency Response Rang of Accelerometers	Sensitivity of Accelerometers	Positions	Sampling Frequency	Sampling Duration
1	0.1~5000 Hz	500 mV/g	Front main bearing	Low/5120 Hz	Long/16 s
2	0.1~5000 Hz	500 mV/g	Rear main bearing	Low/5120 Hz	Long/16 s
3	0.1~5000 Hz	500 mV/g	Outer of the ring gear	Low/5120 Hz	Long/16 s
4	0.5~8000 Hz	100 mV/g	Sun shaft	High/25,600 Hz	Short/4 s
5	0.5~8000 Hz	100 mV/g	Intermediate shaft	High/25,600 Hz	Short/4 s
6	0.5~8000 Hz	100 mV/g	High-speed shaft	High/25,600 Hz	Short/4 s
7	0.5~8000 Hz	100 mV/g	Drive end of the generator	High/25,600 Hz	Short/4 s
8	0.5~8000 Hz	100 mV/g	Nondrive end of the generator	High/25,600 Hz	Short/4 s

## Data Availability

Data available on request due to restrictions eg privacy or ethical.
